# Vitamin E δ-tocotrienol sensitizes human pancreatic cancer cells to TRAIL-induced apoptosis through proteasome-mediated down-regulation of c-FLIP_s_

**DOI:** 10.1186/s12935-019-0876-0

**Published:** 2019-07-22

**Authors:** Rony A. Francois, Anying Zhang, Kazim Husain, Chen Wang, Sean Hutchinson, Michael Kongnyuy, Surinder K. Batra, Domenico Coppola, Said M. Sebti, Mokenge P. Malafa

**Affiliations:** 1 Gastrointestinal Oncology Program, Department of Gastrointestinal Oncology, H. Lee Moffitt Cancer Center and Research Institute, 12902 Magnolia Drive, Tampa, FL 33612 USA; 2 Department of Life Science and Technology, University of Electronic Science and Technology of China, Chengdu, China; 3 Department of Breast Oncology, Tianjin Medical University Cancer Institute and Hospital, Tianjin, China; 4 Department of Biochemistry and Molecular Biology, College of Medicine, University of Nebraska Medical Center, Omaha, NB USA; 5 Department of Anatomical Pathology, H. Lee Moffitt Cancer Center and Research Institute, Tampa, FL USA; 6 Drug Discovery Program, H. Lee Moffitt Cancer Center and Research Institute, Tampa, FL USA

**Keywords:** δ-Tocotrienol, Pancreatic cancer, Apoptosis, TRAIL, c-FLIP

## Abstract

**Background:**

Vitamin E δ-tocotrienol (VEDT), a vitamin E compound isolated from sources such as palm fruit and annatto beans, has been reported to have cancer chemopreventive and therapeutic effects.

**Methods:**

We report a novel function of VEDT in augmenting tumor necrosis factor-related apoptosis-inducing ligand- (TRAIL-) induced apoptosis in pancreatic cancer cells. The effects of VEDT were shown by its ability to trigger caspase-8-dependent apoptosis in pancreatic cancer cells.

**Results:**

When combined with TRAIL, VEDT significantly augmented TRAIL-induced apoptosis of pancreatic cancer cells. VEDT decreased cellular FLICE inhibitory protein (c-FLIP) levels without consistently modulating the expression of decoy death receptors 1, 2, 3 or death receptors 4 and 5. Enforced expression of c-FLIP substantially attenuated VEDT/TRAIL-induced apoptosis. Thus, c-FLIP reduction plays an important part in mediating VEDT/TRAIL-induced apoptosis. Moreover, VEDT increased c-FLIP ubiquitination and degradation but did not affect its transcription, suggesting that VEDT decreases c-FLIP levels through promoting its degradation. Of note, degradation of c-FLIP and enhanced TRAIL-induced apoptosis in pancreatic cancer cells were observed only with the anticancer bioactive vitamin E compounds δ-, γ-, and β-tocotrienol but not with the anticancer inactive vitamin E compounds α-tocotrienol and α-, β-, γ-, and δ-tocopherol.

**Conclusions:**

c-FLIP degradation is a key event for death receptor-induced apoptosis by anticancer bioactive vitamin E compounds in pancreatic cancer cells. Moreover, VEDT augmented TRAIL inhibition of pancreatic tumor growth and induction of apoptosis in vivo. Combination therapy with TRAIL agonists and bioactive vitamin E compounds may offer a novel strategy for pancreatic cancer intervention.

**Electronic supplementary material:**

The online version of this article (10.1186/s12935-019-0876-0) contains supplementary material, which is available to authorized users.

## Background

Pancreatic ductal adenocarcinoma represents a major healthcare problem, with a high fatality rate and increasing incidence, and is likely to increase from the fourth- to the second-leading cause of cancer deaths in the US by 2030 [1–4]. Escape from apoptosis (programed cell death) is a characteristic of pancreatic cancer cells, contributing to their resistance to currently available interventions [5, 6]. Therefore, targeting apoptosis resistance is an important strategy to improve pancreatic cancer outcomes.

Apoptosis is controlled by 2 major signaling pathways: the extrinsic pathway, initiated by death receptors, and the intrinsic pathway, initiated via the mitochondria [7, 8]. Crosstalk between these 2 pathways is mediated by signaling involving the proapoptotic protein Bid [9]. The activation of the extrinsic death receptor-mediated apoptosis pathway relies on a death ligand (e.g., tumor necrosis factor-related apoptosis-inducing ligand [TRAIL]) binding to its corresponding death receptors [10]. Activation of the extrinsic death receptor-mediated apoptotic pathway has received attention as a potential strategy for cancer treatment because its activation preferentially induces apoptosis in transformed or malignant cells but not in most normal cells. Recombinant human TRAIL and the agonist antibodies against death receptor 4 (DR4) and death receptor 5 (DR5), which directly activate extrinsic apoptotic pathways, are being tested in early-phase clinical trials. However, recent studies have shown that many types of cancer cells, including pancreatic ductal adenocarcinoma cells, are resistant to the apoptotic effects of TRAIL [11–13], suggesting that treatment with TRAIL alone may not be sufficient for treating pancreatic cancer. Therefore, sensitizers capable of overcoming TRAIL resistance in pancreatic cancer cells are needed to establish more effective TRAIL-based pancreatic cancer therapies.

Tocotrienols (α-, β-, δ-, and γ-) are 1 of 2 groups of compounds that constitute the vitamin E family [14]. The β-, δ -, and γ-tocotrienols have shown promising efficacy in preventing and treating human cancers, including pancreatic cancer [15–18]. Vitamin E delta-tocotrienol (VEDT) is undergoing early-phase human clinical trials for pancreatic cancer intervention. VEDT exhibits pleiotropic pancreatic anticancer effects, including induction of cell cycle arrest and apoptosis [16, 19] and prevention of angiogenesis and invasion [20–22], demonstrating VEDT’s potential utility as a pancreatic anticancer drug. VEDT also augments gemcitabine activity in pancreatic cancer, suggesting its potentiality as an adjunct in combination therapy. Multiple targets, including p27, K-ras, p53, NF-kB, and VEGF, have been proposed to explain the anticancer effects of VEDT [16, 19–22], but the underlying molecular mechanisms have not yet been fully elucidated.

Here, we show for the first time that VEDT effectively sensitizes pancreatic cancer cells but not human pancreatic ductal epithelial cells to TRAIL-induced apoptosis, suggesting that this combined treatment may provide a safe and effective therapeutic strategy against pancreatic cancer. Furthermore, we provide novel evidence that the prominent sensitizing effect of VEDT on TRAIL-induced apoptosis is primarily through inducing cellular FLICE inhibitory protein (c-FLIP) degradation. Thus our findings highlight a novel mechanism by which VEDT modulates apoptosis in human cancer cells.

## Methods and materials

### Ethics statement

All experiments were carried out in accordance with guidelines set by the Animal Experimental Ethics Committee.

### Chemicals and reagents

Vitamin E analogs α-, β-, γ-, and δ-tocopherols and tocotrienols were kindly gifted by Davos Life Sciences (Helios, Singapore). Ethanol was purchased from Aaper Alcohol & Chemical (Shelbyville, KY). Recombinant human TRAIL was purchased from Invitrogen (Carlsbad, CA). FLICE inhibitory protein (FLIP) antibody (NF6) was purchased from Alexis Biochemicals (Enzo Life Sciences, Farmingdale, NY). DR5, DR4, DCR1, DCR2, and DCR3 antibodies were purchased from Abcam (Cambridge, MA). Caspase-3 (#9662), caspase-8 (#9746), PARP (#9542), cleaved caspase 3 (#9661S), cleaved caspase 8 (#9496), cleaved PARP (#5625), and green fluorescence protein (GFP) antibodies were purchased from Cell Signaling Technology (Beverly, MA). Hemagglutinin- (HA-) probe antibody, protein A/G PLUS-Agarose, and secondary antibodies were purchased from Santa Cruz Biotechnology (Santa Cruz, CA). All cDNA plasmids were purchased from Origene (Rockville, MD). The sulforhodamine B- (SRB-) based In Vitro Toxicology Assay Kit and all other reagents were purchased from Sigma-Aldrich (St. Louis, MO) unless otherwise specified.

### Cell lines and culture conditions

AsPc-1, BxPc-3, MiaPaCa-2, PANC-1, and SW1990 human pancreatic cancer cell lines (American Type Culture Collection, Manassas, VA) were cultured in complete 1 × Dulbecco’s Modified Eagle Medium (DMEM; Invitrogen) supplemented with 10% fetal bovine serum (HyClone, Logan, UT), penicillin–streptomycin (Mediatech, Herndon, VA) at a final concentration of 50 IU/mL (penicillin), 50 mg/mL (streptomycin), and 2 mM l-glutamine (Mediatech). AsPc-1 and BxPc-3 cells were cultured in complete RPMI 1640 medium (Invitrogen) containing 10% fetal bovine serum, penicillin–streptomycin, 10 mM HEPES buffer (Mediatech), 1 mM sodium pyruvate (Invitrogen), and 2 mM l-glutamine (Mediatech). HPDE6-C7 human pancreatic ductal epithelial cells (gift from G. Springett, Moffitt Cancer Center) were cultured in keratinocyte serum-free medium (Invitrogen) containing the provided epidermal growth factor and bovine pituitary extract supplements. Immortalized human pancreatic normal epithelial (HPNE) cells (gift from P. Campbell, Moffitt Cancer Center) were cultured in a mixture of DMEM and M3:F media (INCELL, San Antonio, TX) at a ratio of 3 parts DMEM and 1 part M3:F media and were supplemented with 5% fetal bovine serum. All cell lines were maintained at 37 °C in a humidified incubator with 5% CO_2_. Cells were passaged regularly with .05% trypsin-ethylenediaminetetraacetic acid (Invitrogen) to maintain logarithmic-phase growth. For all experiments, cells were gently detached with Accutase enzyme cell detachment medium (eBioscience, San Diego, CA) in accordance with the provided protocol, with Trypan Blue and hemacytometer used to determine viable cell number.

### Cell viability and cell survival assay

Cells were seeded in 96-well plates at a density of 3000 cells/well and allowed to attach overnight. After cells were incubated for 72 h with various concentrations of drugs (10^−5^ to 10^−4^ M) or ethanol (< 5%) as vehicle control, media were aspirated and replaced with 20 µL of 1 mg/mL MTT (3-[4,5-dimethylthiazol-2-yl]-2,5-diphenyltetrazolium bromide) and incubated for 2 to 4 h at 37 °C in a humidified atmosphere of 5% CO_2_. Media were aspirated, 200 µL of dimethyl sulfoxide were added to each well, plates were incubated for 5 min with shaking, and absorbance was read at 540 nm. Cell survival was determined using the SRB colorimetric assay for cytotoxicity in accordance with manufacturer’s instructions. The SRB assay is an established surrogate for cell survival [23]. Absorbance was recorded using a microplate reader at 565 nm.

### Determination of apoptosis

Cells (1 × 10^6^) were seeded in 100-mm tissue culture dishes and allowed to adhere overnight. Cells were treated the following day at varying doses with δ-tocotrienol and collected at specific time points to assess apoptosis by either terminal deoxynucleotidyl transferase-mediated nick end labeling (TUNEL) or annexin V staining. Cells were washed with phosphate-buffered saline (PBS), pelleted, and counted using a hemacytometer. For all TUNEL experiments, ~ 1 × 10^5^ cells were fixed onto a glass slide using a Cytospin III centrifuge (Thermo Shandon Inc., Pittsburgh, PA) and then fixed in 4% paraformaldehyde in PBS solution overnight at 4 °C. Cells were made permeable the next day via incubation in 0.1% sodium citrate-0.1% Triton X-100 solution at 4 °C for 2 min and then labeled for apoptotic DNA strand breaks using the in situ cell death detection kit, AP (Roche Applied Science, Indianapolis, IN) in accordance with manufacturer’s instructions. Cells were stained in Vectashield mounting medium (Vector Laboratories, Burlingame, CA) containing DAPI (4,6-diamidino-2-phenylindole) to counterstain DNA. Fluorescein-labeled DNA strand breaks (TUNEL-positive cells) were then visualized using a fluorescence microscope (Leica Microsystems Inc., Bannockburn, IL), and 5 representative pictures of the slide field were taken with a digital camera (Diagnostic Instruments, Inc., Sterling Heights, MI). TUNEL-positive nuclei (green) were scored and compared with DAPI-stained nuclei (blue) to determine the induction percentage of apoptosis for each treatment group. Annexin V and propidium iodide staining were performed with the Annexin V-FITC Apoptosis Detection Kit I (BD Biosciences) as follows: pelleted cells were washed twice with cold PBS and then briefly resuspended in a 1× binding buffer before incubation with annexin V-FITC and propidium iodide according to the manufacturer’s instructions. Flow cytometry was performed using a FACScan flow cytometer (Becton–Dickinson), with analysis using FLOW-JO software (Tree Star, Ashland, OR) to assess the Annexin-positive cell population.

### Caspase enzymatic activity assay and use of irreversible caspase inhibitors

Enzymatic activities of caspases-3, -8, and -9 were determined using each respective fluorogenic substrate (Calbiochem, EMD Biosciences, San Diego, CA). Protein extract (20 µg) was incubated in a reaction buffer containing 50 mM Tris (pH 7.5) and caspase substrate at a final concentration of 20 µM in 96-well plates. After 3-h incubation at 37 °C, liberated fluorescent 7-amido-4-methyl-coumarin groups were quantified using a multi-well plate VersaFluorTM Fluorometer with an excitation filter of 380 nm and an emission filter of 460 nm (Bio-Rad Laboratories, Hercules, CA). When irreversible inhibitors of caspases-3, -8, and -9 were used (Calbiochem, EMD Biosciences), each inhibitor was used at a final concentration of 10 µM and incubated with the treatment group 4 h before treatment with tocotrienol. Tocotrienol was then added to the media containing the inhibitor at the indicated final concentration and treated for the desired length of time.

### Western blot analysis

To prepare whole cell protein lysates, cells were seeded at 1 × 10^6^ cells per 100-mm tissue culture dish and treated the following day with δ-tocotrienol. After desired treatment times, cells were collected, washed with PBS, pelleted, lysed on ice for 5 min, and then stored at − 20 °C. Tumor tissues were cut into small pieces and homogenized with tissue protein extraction reagent (T-PER, Pierce) and centrifuged at 10 000 RPM for 10 min. The extracted proteins were stored at − 20 °C. Protein concentration was determined to ensure equal protein loading. Protein extracts were resolved using a 12.5% SDS-PAGE gel and then transferred to nitrocellulose membrane. Protein separation was briefly assessed using Ponceau S solution (Pierce). Membranes were then washed with fresh Tris-buffered saline containing .1% Tween 20 (1× TBS-T) and blocked with 5% nonfat dry milk solution. Primary antibodies were incubated in 3% BSA solution either overnight at 4 °C or for 1 h at 25 °C. When reblotting was necessary, antibodies were stripped from membrane by incubating with Western blot stripping buffer (Pierce) for 0.5 h at 25 °C. High-affinity antibodies were stripped from membrane by washing for 5 min with a 0.2 M NaOH solution 3 times at 25 °C, followed by 3 × 5-min washes with deionized water. Membranes were thoroughly washed in 1× TBS-T at 25 °C and incubated with secondary antibody (horseradish peroxidase-conjugated) at 1:2000 dilutions for 1 h at room temperature. The washed blot was then treated with SuperSignal West Pico chemiluminescent substrate (Pierce Biotech) for positive antibody reaction. Membranes were exposed to x-ray film for visualization and densitometric quantization of protein bands using AlphaEaseFC software (Alpha Innotech, Santa Clara, CA).

### Generation of MiaPaCa-2 cells stably expressing GFP-labeled c-FLIP_s_

MiaPaCa-2 cells were transfected with a plasmid-containing full-length open-reading frame cDNA of c-FLIP_s_ fused with GFP at the C-terminus in a pCMV6-AC-GFP vector. MiaPaCa-2 cells transfected with the pCMV6-AC-GFP vector containing GFP alone served as control. All transfections were performed using Lipofectamine™ 2000 reagent (Invitrogen) at a 1 µg:1 µL DNA-to-Lipofectamine 2000 ratio, per manufacturer’s instructions. Cells were selected for 2 weeks with G418 sulfate at a final concentration of 500 µg/mL.

### Immunoprecipitation for detection of c-FLIP ubiquitination

Mia-FLIP_s_ cells, stably expressing GFP-FLIP_s_, were transfected with HA-ubiquitin plasmid (Addgene) using the Lipofectamine 2000 transfection reagent, in accordance with the manufacturer’s instructions. After 24 h, cells were treated with δ-tocotrienol alone (50 µM) or in combination with MG-132 (25 µM) for 6 h and then lysed for immunoprecipitation of ubiquitin-bound c-FLIP_s_, using GFP polyclonal antibody (Abcam). Harvested cells were washed 3 times with ice-cold PBS and resuspended in 1 mL of ice-cold CelLytic™ M cell lysis reagent (Sigma) containing protease inhibitors. Cleared lysates were then incubated with 10 µg of GFP polyclonal antibody and 100 μL of 50% slurry of Protein A/G Agarose (Santa Cruz, sc-2003) into the lysate, and the mixture was rotated overnight at 4 °C. Beads were washed three times with lysate buffer followed by spinning for 5 s at 10,000 *g*. After the washes, 50 μL of 1 × sample buffer was added to the bead pellet and boiled at 100 °C for 5 min followed by the detection of ubiquitin-bound c-FLIP_s_ with Western blotting using anti-HA antibody (Santa Cruz, sc-57592).

### Quantitative PCR analysis

RNA was isolated from cells using the AllPrep RNA/Protein kit from Qiagen (Germantown, MD), in accordance with the manufacturer’s instructions. RNA extracts were analyzed by quantitative PCR by the Molecular Biology Core at the Moffitt Cancer Center using the TaqMan gene expression assay kit to assess c-FLIP mRNA levels. 18S rRNA was used as an internal control.

### In vivo tumor xenograft model

MiaPaCa-2 cells were harvested and resuspended in fresh media, and the number of viable cells was determined. Cells were then injected subcutaneously into flanks of NIH-III nude SCID mice (Charles River Laboratories, Boston, MA) at a density of 1 × 10^6^ cells/per flank in a 1:1 solution of PBS to Matrigel Matrix (BD BioSciences). Mice were randomized into control and treated groups, with controls receiving ethanol-extracted olive oil (vehicle control) alone and treated mice receiving 200 mg/kg of δ-tocotrienol in vehicle. All treatments were administered orally by gavage 12 times/week (weekdays, 2×/day; weekends, 1×/day), and mouse weights were recorded twice per week.

For the drug combination (TRAIL and VEDT) experiment, female Athymic nude mice (n = 20) were injected subcutaneously with MiaPaCa-2 cells into flanks at a density of 1 × 10^6^ cells/per flank in a 1:1 solution of PBS to Matrigel Matrix. Mice were randomized into 4 groups of 5 animals each and treated as follows: (Group 1) Vehicle control mice were given orally ethanol-extracted olive oil twice a day and intraperitoneal (IP) injection of PBS on alternate days for 4 weeks, (Group 2) TRAIL mice were injected with TRAIL 20 µg/kg IP on alternate days for 4 weeks, (Group 3) VEDT mice were given orally VEDT (200 mg/kg) twice a day for 4 weeks, and (Group 4) TRAIL + VEDT mice were injected with TRAIL 20 µg/kg IP on alternate days and VEDT orally (200 mg/kg) twice a day for 4 weeks. Tumors were measured daily with calipers using the formula T = L × W × [(L + W)/2] × 0.5236, where T is tumor volume, L is smallest tumor diameter, and W is largest tumor diameter. Animal data are representative of 2 independent experiments. Animals were sacrificed after 4 weeks and tumor weights were recorded and half tumor tissues were fixed in buffered formalin for histology staining and the other half were immersed in liquid nitrogen then frozen at − 80 °C for further biochemical analyses.

### Histological and immunohistochemical analyses

At the end of the study, mice were killed, and tumors were extracted and embedded in paraffin sections for further analyses. Immunohistochemistry was performed using the Ventana Discovery XT automated system (Ventana Medical Systems, Tucson, AZ), per manufacturer’s protocol, with proprietary reagents. Slides were deparaffinized on the automated system with EZ Prep solution. Sections were heated for antigen retrieval. For immunohistochemistry, tissue sections were incubated with antibodies. Detection was performed using the Ventana OmniMap kit. Apoptosis by TUNEL staining, cleaved caspase-8, cleaved caspase-3, and Ki-67 staining and hematoxylin and eosin staining were quantified by the Moffitt Anatomic Pathology Core.

### Statistical analyses

Data, expressed as mean ± standard error of the means, were analyzed statistically using unpaired t-tests or 1-way analysis of variance (ANOVA), as appropriate. ANOVA was followed by Duncan’s multiple range tests using SAS statistical software for comparisons between different treatment groups. Analyses of in vivo data were performed using the GraphPad Prism program (GraphPad Software, San Diego, CA) on the entire tumor growth curve using a 2-way ANOVA, mixed-effect model for repeated measures, with respect to the column factor corresponding to δ-tocotrienol treatment. Statistical significance was set at *P *< .05.

## Results

### VEDT inhibits the growth of human pancreatic cancer cells in vitro and in vivo and induces apoptosis

**Fig. 1:**
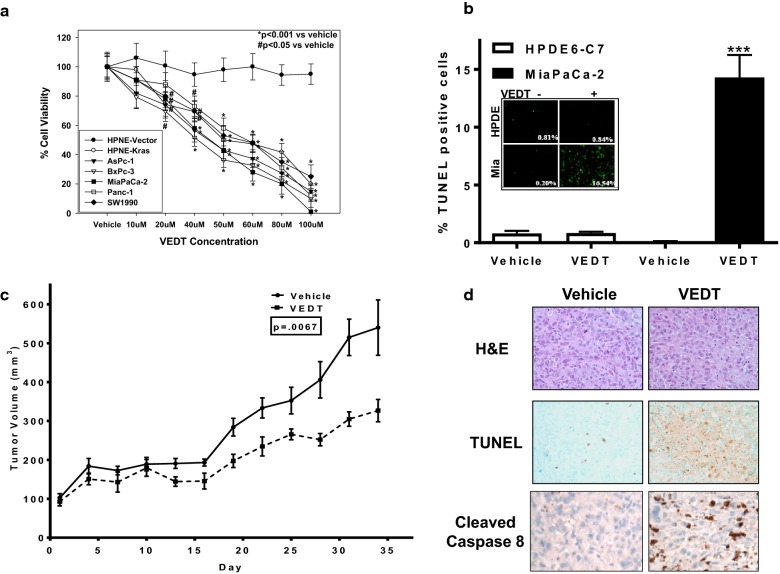
The effects of VEDT on survival, apoptosis induction, and tumor tissues in mice. **a** Effect of VEDT dose response on survival at 72 h in panel of human pancreatic cancer cell lines (AsPc-1, BxPc-3, MiaPaCa-2, Panc-1, and SW1990) and a pair of immortalized HPNE containing vector alone (HPNE-V) or transformed with oncogenic KRAS (HPNE-KRAS). Points, means; bars, standard error (n = 3–5, *P < .001, ^#^*P *< .05). **b** Effect of δ-tocotrienol (VEDT; 20 µM) on induction of apoptosis, as measured by TUNEL staining in HPDE6-C7 immortalized human pancreatic ductal epithelial cells and MiaPaCa-2 pancreatic cancer cells. Ethanol served as vehicle control. VEDT significantly induced apoptosis in MiaPaCa-2 cells. Columns, means; bars, standard deviation (n = 4, ***P < .001). **c** Growth inhibition of subcutaneous MiaPaCa-2 xenografts in NIH-III mice treated orally twice daily with 200 mg/kg VEDT. Olive oil served as vehicle control. Points, means; bars, standard deviation (n = 5, *P *< .007). **d** Effect of VEDT on apoptosis (TUNEL) and cleaved caspase 8 in formalin-fixed, paraffin-embedded MiaPaCa-2 xenograft tumor tissues in mice. H&E, hematoxylin and eosin. VEDT significantly induced apoptosis (*P *< .05) compared with vehicle-treated mice (n = 5)

We first examined whether VEDT alone could inhibit the survival of a panel of human pancreatic cancer cells. Immortalized HPNE cells and HPNE cells transformed with oncogenic K-Ras (HPNE K-Ras) served as controls. VEDT significantly inhibited the viability of 5 human pancreatic cancer cell lines and the HPNE K-Ras cell line in a dose-dependent manner. In contrast, HPNE cells were markedly less sensitive to VEDT (Fig. 1a).

Because decreased cell survival may be caused by many processes (i.e., autophagy, anoikis, apoptosis, necrosis), we next investigated whether VEDT survival inhibition could be attributed to increased apoptosis. Using TUNEL staining, which detects apoptotic DNA fragmentation, we examined VEDT’s ability to induce apoptosis in MiaPaCa-2 cells. VEDT administration resulted in a statistically significant (*P *< .001) induction of apoptosis in MiaPaCa-2 cells (Fig. 1b). In contrast, normal HPDE6-C7 cells were spared from apoptotic cell death at the equivalent dose of 20 µM, indicating that VEDT selectively exhibits activity against malignantly transformed cells. To verify these findings in vivo, we examined the proapoptotic activity of VEDT in a human pancreatic cancer xenograft mouse model, as described previously [16]. MiaPaCa-2 cells were injected subcutaneously into NIH-III nude mice and allowed to reach a volume of 100 mm^3^. Immunohistochemistry of paraffin-embedded tumors of treated mice showed that VEDT not only caused a statistically significant inhibition of tumor growth compared with vehicle (*P *< .007) but also resulted in significant (*P *< .05) induction of apoptosis in tumor tissues (Fig. 1c, d).

#### VEDT induces caspase-8-dependent apoptosis in human pancreatic cancer cells

**Fig. 2:**
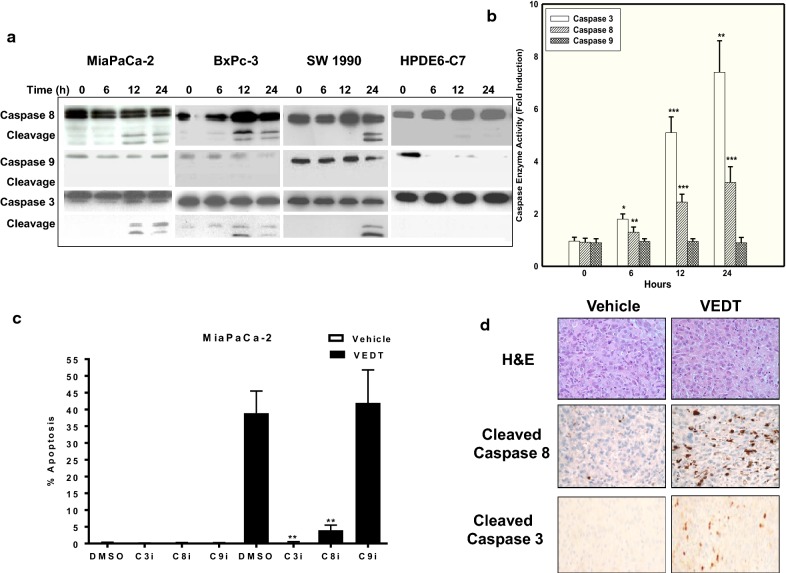
Western blot analyses of caspases. **a** Western blot analyses of caspases (casp) -3, -8, and -9, cleavage in MiaPaCa-2, BxPc-3, SW1990 pancreatic cancer cells, and HPDE6-C7 immortalized human pancreatic ductal epithelial cells treated with VEDT for 0, 6, 12, or 24 h. β-Actin served as loading control. **b** Enzymatic activities of caspases-3, -8, and -9 in MiaPaCa-2 cells following VEDT treatment at indicated time points. Columns, means; bars, standard deviation (n = 4, *P < .05, **P < .01, and ***P < .001). **c** Effect of VEDT induction of apoptosis by pretreatment with covalent irreversible inhibitors of caspases-3, -8, and -9 (C3i, C8i, and C9i). Dimethyl sulfoxide served as vehicle control for each inhibitor. *NS* not significant. Columns, means; bars, standard deviation (n = 4, **P < .01 compared with untreated cells). **d** Immunohistochemical analysis of cleaved caspase-8 and cleaved caspase 3 in MiaPaCa-2 xenograft tumors. H&E, hematoxylin and eosin. VEDT treatment increased cleaved caspase-8 staining compared to vehicle (n = 5)

**Fig. 3:**
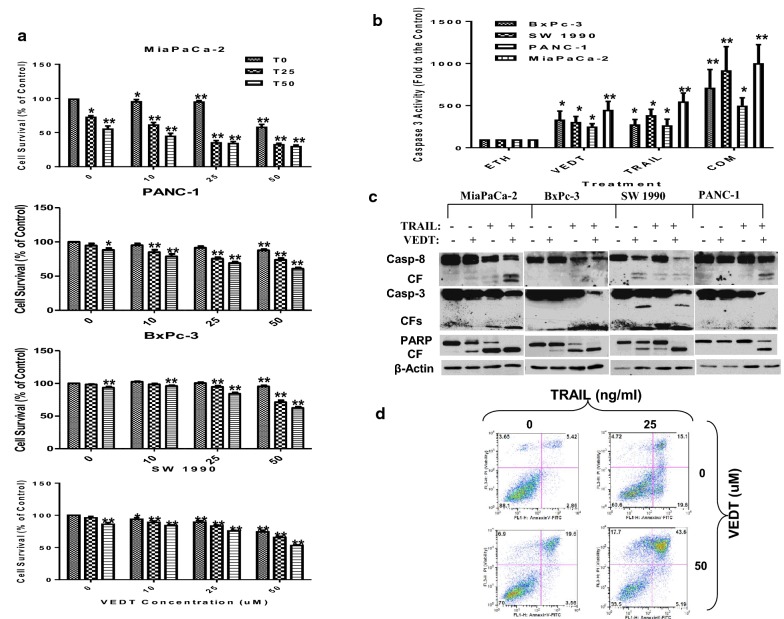
The effects of TRAIL and VEDT on cell survival. **a** Effect of TRAIL alone (25 and 50 ng/mL) or in combination with VEDT at multiple concentrations (10, 25, 50 μM) on cell survival at 24 h in panel of human pancreatic cancer cell lines. Columns, means; bars, standard deviation (n = 3, *P < .05 and **P < .01). **b** Effect of TRAIL alone or in combination with VEDT on the enzymatic activity of caspase-3 in panel of human pancreatic cancer cell lines after 24 h. Columns, means; bars, standard deviation (n = 3, *P < .05 and **P < .01). **c** Western blot analysis of cleavage of caspase-8, caspase-3, and PARP (CF = cleaved fragment) in a panel of human pancreatic cancer cell lines following treatment with TRAIL alone or in combination with VEDT for 24 h (n = 3). β-Actin served as loading control. **d** Flow cytometry analysis of apoptosis as measured by annexin V staining following treatment with TRAIL alone (25 ng/mL) or in combination with VEDT (50 μM) in MiaPaCa-2 cells (n = 3). In annexin V assay, percentages of positive cells in the top right and bottom right quadrants were added to get the total apoptotic cells. *P < .001 compared with untreated cells; **P < .01 compared with untreated cells

To determine the mechanism by which VEDT induces apoptosis in pancreatic cancer cells, we measured VEDT’s effects on the activation of intracellular caspase cascades and the dependence of VEDT-induced apoptosis on caspase activation. Cleavage of procaspase-8 and procaspase-3 were detected in VEDT-treated MiaPaCa-2, BxPC-3, and SW1990 cell lines. Although procaspase-9 was detected in these cells, cleavage of procaspase-9 was not detected (Fig. 2a). Consistent with the immunoblot detection, caspase-8 and caspase-3 enzyme activities were both increased from 6 to 24 h following VEDT treatment in MiaPaCa-2 cells; however, caspase-9 activity was not significantly altered after VEDT treatment (Fig. 2b). Furthermore, as shown in Fig. 3c, inhibition of caspase-8 and caspase-3 activation using Z-Ile-Glu(OMe)-Thr-Asp(OMe)-CH2F and Ac-Asp-Glu-Val-Asp-CMK resulted in significant inhibition of VEDT-induced apoptosis in MiaPaCa-2 cells, whereas inhibition of caspase-9 activation using Z-Leu-Glu(OMe)-His-Asp(OMe)-CH2F resulted in no effect on VEDT-induced apoptosis in these cells. Moreover, significant induction of cleaved caspase-3 was also detected in MiaPaCa-2 xenograft tumors treated with VEDT (Fig. 2d). Thus, these results collectively show that VEDT induces caspase-8-dependent apoptosis.

### VEDT augments TRAIL-mediated apoptosis in human pancreatic cancer in vitro and in vivo

**Fig. 4:**
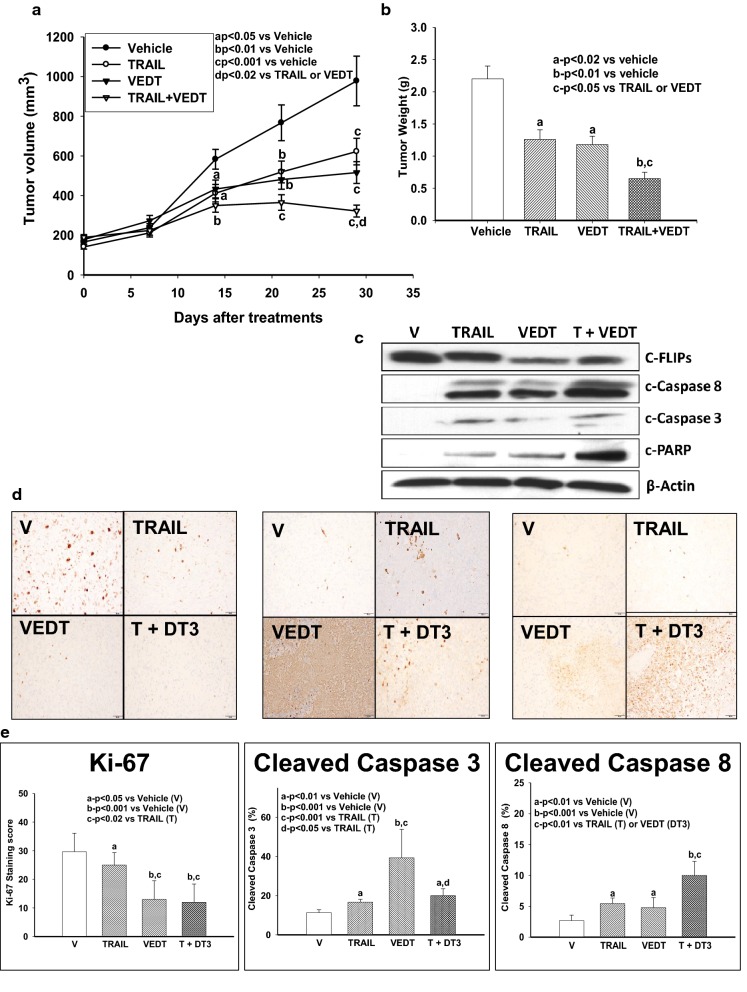
The effects of TRAIL and VEDT on tumor volume. **a** Effect of TRAIL (20 µg/Kg intraperitoneally) on alternate days and VEDT (200 mg/kg, Orally) twice a day and combination of the two drugs for 4 weeks on MiaPaPa-2 tumor volume in Athymic nude mice. The tumor volume significantly reduced in TRAIL and VEDT treated mice for 4 weeks compared to vehicle (^c^*P* < .001 and ^c^*P* < .001). However the combination of the two drugs resulted in a more significant inhibition of tumor volume compared to vehicle (^c^*P *< .001) or either drug alone (^d^*P *< .02). **b** Effect of TRAIL (20 µg/Kg intraperitoneally) on alternate days and VEDT (200 mg/kg, Orally) twice a day and combination of the two drugs for 4 weeks on MiaPaPa-2 tumor weight in athymic nude mice. The tumor weights were significantly reduced in TRAIL and VEDT (^a^*P *< .02) treated mice for 4 weeks compared to vehicle (^a^*P *< .02 and ^a0^*P* < .02). However, the combination of the two drugs resulted in a more significant inhibition of tumor weight compared to vehicle (^b^*P* < .01) or either drug alone (^c^*P* < .05). **c** Western blot analyses of cleaved caspase-8, cleaved caspase-3, cleaved PARP, and c-FLIPs in tumor tissues following treatment with TRAIL alone or in combination with VEDT for 4 weeks (n = 5). β-Actin served as loading control. TRAIL and VEDT alone induced apoptosis (elevated cleaved caspase-8, cleaved caspase-3, and cleaved PARP). Greater induction of apoptosis was noted in tumor tissues following combined drug treatment than with either drug alone. VEDT alone and in combination with TRAIL but not TRAIL alone depleted tumor tissue c-FLIP_s_ expression. **d** Histological data depicting the Ki-67, cleaved caspase-3, and cleaved caspase-8 in tumor tissues following treatment with TRAIL (T) alone or in combination with VEDT (DT3) for 4 weeks (n = 5). **e** Greater and significant induction of apoptosis (cleaved caspase-8, ^b^*P* < .001) and inhibition of tumor cell proliferation (Ki-67, ^b^*P *< .001) was noted in tumor tissues following combined drug treatment than with either drug alone. VEDT alone significantly induced cleaved caspase 3 compared to vehicle (^b^*P* < .001) and was greater than TRAIL-alone treatment (^c^*P* < .001). The combination significantly induced cleaved caspase 3 compared to vehicle (^a^*P *< .01) and compared to TRAIL alone (^d^*P* < .05)

If VEDT-induced apoptosis is caspase-8-dependent, we speculated that adding exogenous recombinant TRAIL to VEDT treatment would result in enhanced apoptosis induction. To test this hypothesis, we treated 4 human pancreatic cancer cell lines (MiaPaCa-2, BxPC-3, SW1990, and Panc-1) with VEDT alone, TRAIL alone, or both drugs combined and then accessed cell survival, caspase-3 enzyme activity, and apoptosis. As presented in Fig. 3a, the combination of VEDT at concentrations of 10 to 50 μM with TRAIL (25 or 50 ng/mL) was more effective in decreasing tumor cell number than either agent alone. For example, in MiaPaCa-2, both VEDT alone (at 25 μM) and TRAIL alone (at 25 ng/mL) decreased cell numbers by 15% and 30%, respectively. However, combining the 2 agents reduced cell numbers by > 50%; more than the sum of each agent’s effects alone. Measuring caspase-3 enzyme activity further highlights the effects of combining VEDT and TRAIL on apoptosis induction. Figure 3b shows that the combination of 50 μM VEDT and 25 ng/mL TRAIL more effectively activated caspase-3 enzyme activity than either agent alone in all pancreatic cancer cell lines examined. For example, in SW1990 cells, both VEDT alone and TRAIL alone increased enzyme activity by 250- and 300-fold over controls; however, the 2 agents combined increased enzyme activity by > 1000-fold, which was greater than the sum of the effects of each agent alone. Using Western blotting, we also detected the strongest bands of cleaved caspases and poly ADP ribose polymerase (PARP) or the most reduction of procaspases and PARP in cells exposed to VEDT + TRAIL compared with VEDT or TRAIL alone (Fig. 3c). Moreover, we used annexin V staining to detect apoptosis in MiaPaCa-2 and Panc-1 cells exposed to VEDT + TRAIL. During 24-h treatment, VEDT + TRAIL combined was much more effective in inducing apoptosis (44% and 49%) than TRAIL (15% and 23%) or VEDT (20% and 25%) alone (Fig. 3d and Additional file 1: Figure S1). Collectively, these results clearly indicate that VEDT synergized with TRAIL to augment induction of apoptosis in human pancreatic cancer cells. To verify these findings in vivo, we examined VEDT’s antitumor and proapoptotic activity in a human pancreatic cancer xenograft mouse model. MiaPaCa-2 cells were injected subcutaneously into Athymic nude mice and allowed to reach a volume of 100 mm^3^, and the single-drug treatments and the combination were given for 4 weeks. Tumor volume was recorded every week, and after 4 weeks, the tumor weights were recorded and the tumor tissues were processed to analyze c-FLIP and apoptotic protein expression by Western Blot. Tumor volume and weight were significantly reduced in mice previously treated with TRAIL (^c^*P* < .001 and ^a^*P *< .02) and VEDT (^c^*P *< .001 and ^a^*P *< .02) for 4 weeks compared to those treated with vehicle (Fig. 4a, b). However, the combination of the 2 drugs resulted in a more significant inhibition of tumor volume (^c^*P* < .001) and weight (^b^*P* < .01) than for tumors treated with vehicle or either drug alone (^d^*P* < .02 and ^c^*P* < .05). The Western blot data show that TRAIL and VEDT induced apoptosis (cleaved caspase-8, cleaved caspase-3, and cleaved PARP), and the combination induced greater apoptosis in the tumor tissues than either drug alone (Fig. 4c). VEDT but not TRAIL depleted the c-FLIP expression in tumor tissues compared to vehicle. However, combining the 2 drugs profoundly depleted the c-FLIP expression in tumor tissues (Fig. 4c). The histological data in tumor tissues further confirmed that VEDT significantly enhanced the TRAIL-induced apoptosis (cleaved caspase-8) and inhibition of tumor cell proliferation (Ki-67), as depicted in Figs. 4d, e.

### VEDT induces apoptosis and augments TRAIL activity through down-regulation of c-FLIP_S_ in human pancreatic cancer cells

**Fig. 5:**
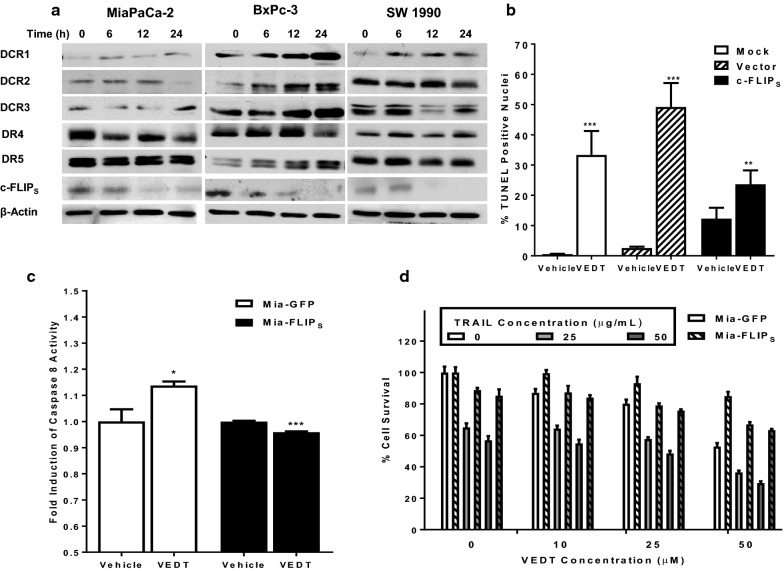
The effect of VEDT on cell death and survival. **a** Time course of VEDT activity on decoy receptors and death receptor pathway (DR4, DR5, DCR1, DCR2, DCR3) and c-FLIP_s_ in panel of pancreatic cancer cells by Western blot (n = 3). **b** Effect of transient ectopic transfection of c-FLIP_s_ in pCMV6-AC-GFP plasmid vector on apoptosis in MiaPaCa-2 cells treated with VEDT for 24 h as measured by TUNEL and quantified by analytic microscopy (inset). Columns, means; bars, standard deviation (n = 3, **P < .01 and ***P < .001). **c** Effect of VEDT on caspase-8 enzymatic activity in Mia-GFP and Mia-FLIP cells. Columns, means; bars, standard deviation (n = 3, *P < .001 and ***P < .05). **d** Effect of VEDT on survival in Mia-GFP and Mia-FLIP cells. Columns, means; bars, standard deviation (n = 3, *P < .001, **P < .01, and ***P < .05)

The mechanisms by which agents augment TRAIL-induced apoptosis include modulation of proteins upstream to caspase-8 activation. These mechanisms include induction of the death receptors DR4 and/or DR5, decreased expression of decoy receptors DCR1, DCR2, and DCR3, and decreased expression of the short isoform of c-FLIP (c-FLIP_s_), which inhibits apoptosis by preventing the recruitment of caspase-8 into the death-inducing signaling complex. Thus we determined whether VEDT modulated DCR1, DCR2, DCR3, DR4, DR5, or c-FLIP_s_ expression in human pancreatic cancer cells by Western blot analysis. We detected a consistent time-dependent decrease in FLIP_s_ expression that preceded VEDT-induced activation of caspase-8 in MiaPaCa-2, BxPC-3, and SW1990 cells exposed to VEDT, which occurred at 6 h and was sustained for at least 16 h (Fig. 5a). In contrast, there was no consistent modulation of DCR1, DCR2, DCR3, DR4, or DR5 by VEDT.

We next investigated whether inhibition of c-FLIP_s_ is required for induction of apoptosis by VEDT using MiaPaCa-2 cells transfected with pCMV6-AV-GFP vector-containing c-FLIP_s_ tagged with GFP. Figure 5b shows that we observed a statistically significant induction of apoptosis in mock and vector-transfected cells treated with VEDT (*P *< .01 and *P *< .001). Importantly, transient overexpression of c-FLIP_s_ not only abrogated VEDT-induced apoptosis compared with vehicle (not significant) but also rescued endogenous c-FLIP_s_ inhibition compared with vector transfection. To further study the role of c-FLIP_s_, we created MiaPaCa-2 cells stably overexpressing c-FLIP_s_ (Mia-FLIP) or GFP vector alone (Mia-GFP; Additional file 2: Figure S2B–E). This enforced ectopic overexpression of c-FLIP_s_ significantly abrogated VEDT induction of caspase-8 enzymatic activity and inhibition of survival (Fig. 5c). Moreover, c-FLIP_s_ overexpression in MiaPaca-2 cells resulted in inhibition of VEDT augmentation of TRAIL-induced inhibition of cell survival in pancreatic cancer cells. For example, TRAIL (25 ng/L) and VEDT (25 μM) treatment of MiaPaCa-2 cells with GFP vector decreased cell numbers by 30% and 20%, respectively, whereas the combination decreased cell number by 45%. In contrast, the same concentration of the agents in MiaPaCa-2 cells expressing c-FLIP_s_ decreased cell numbers by 10% and 5%, respectively, with the combination decreasing cell numbers by only 20% in these cells (Fig. 5d).

### VEDT down-regulates c-FLIP_s_ through promoting ubiquitin/proteasome-mediated degradation

**Fig. 6:**
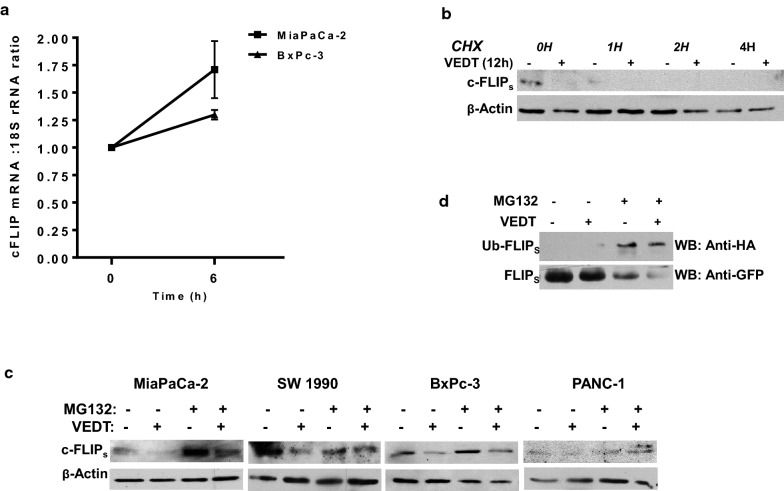
The effect of VEDT on c-FLIP. **a** Effect of VEDT treatment on c-FLIP messenger RNA levels in MiaPaCa-2 and BxPc-3 cells at 6 h. 18S rRNA served as internal control for each sample (n = 3). **b** Half-life of c-FLIP_s_ protein in MiaPaCa-2 cells as determined by cyclohexamide (CHX) (25 µg/ml) pretreatment for the indicated duration, following 12-hour treatment with either vehicle (ethanol) or VEDT (n = 3). **c** Effect of MG132 protease inhibitor on c-FLIP protein levels in presence or absence of VEDT in a panel of pancreatic cancer cell lines (MiaPaCa-2, SW1990, BxPc-3 and Panc-1). The cells were pretreated with MG132 (25 µM) for 6 h followed by VEDT treatment for 24 h (n = 3). **d** Effect of MG132 protease inhibitor on ubiquitin-bound c-FLIP in the presence or absence of VEDT in MiaPaCa-2 cells. The cells were pretreated with MG132 (25 µM) for 6 h followed by VEDT treatment for 24 h (n = 3)

To determine how VEDT regulates c-FLIP_s_, we investigated VEDT’s effect on c-FLIP_s_ transcription and translation. Regulation of c-FLIP_s_ has been shown to occur at the level of transcription and translation, as well as post-transitionally at the protein level [24]. To this end, mRNA levels from VEDT-treated MiaPaCa-2 and BxPc-3 cells were analyzed by quantitative PCR at 6 h, at which point protein levels of c-FLIP_s_ seemed to first decrease. c-FLIP_s_ mRNA levels were normalized to those of 18S rRNA (internal control). At 6 h after VEDT treatment, we observed that c-FLIP_s_ messenger RNA (mRNA) levels had not decreased but had actually increased versus baseline (Fig. 6a).

To validate our findings, we investigated the ability of VEDT to down-regulate c-FLIP_s_ protein expression in the absence of mRNA translation using the protein synthesis inhibitor cyclohexamide. VEDT was found to accelerate c-FLIP_s_ turnover compared with turnover observed with cyclohexamide alone (Fig. 6b). Next, we sought to identify whether proteasomal degradation could be implicated in VEDT down-regulation of c-FLIP_s_. We found that inhibition of c-FLIP_s_ could be prevented by pretreatment with the *pan*-protease inhibitor MG132 in all cell lines tested (Fig. 6c). Because ubiquitination has been identified as an important event associated with proteasomal degradation [25], we next transfected human influenza HA-tagged ubiquitin into MiaPaCa-2 cells stably overexpressing GFP-tagged c-FLIP_s_. Using co-immunoprecipitation techniques, we evaluated the fraction of ubiquitin-bound c-FLIP_s_. As indicated in Fig. 6d, treatment with VEDT resulted in a marked shift in the fraction of ubiquitin-bound c-FLIP_s_ in the presence of MG-132 when compared to that of MG-132 alone. Collectively, these data support the concept that VEDT decreased c-FLIP levels through promoting ubiquitin/proteasome-mediated degradation of c-FLIP.

#### Potencies of vitamin E compounds in inhibiting cell survival and enhancing cell death receptor-mediated apoptosis are associated with their abilities to down-regulate c-FLIP

We previously demonstrated that VEDT is the most bioactive of all of the naturally occurring vitamin E analogs (Additional file 3: Figure S3A) [16]. Here, we report that VEDT is the most powerful vitamin E inhibitor of cell survival and c-FLIP protein expression in pancreatic cancer cells. Of note, c-FLIP down-regulation was directly associated with the ability of each vitamin E compound to inhibit cell survival (Additional file 3: Figure S3B, C). In addition, VEDT also augmented TRAIL-induced cell death in HPNE-Kras cells but not in normal HPNE-vector cells (Additional file 2: Figure S2A).

## Discussion

Pancreatic cancer is highly aggressive, with only 6% of patients surviving 5 years after diagnosis. Suppression of programed cell death is a hallmark of this cancer, which it displays as increased viability and resistance to cytotoxic therapies [26]. One example of this resistance to therapy is pancreatic cancer’s varied sensitivity to TRAIL, a tumor-selective cytokine, which activates the extrinsic apoptotic pathway [27–31]. Therefore, there is an important need for proapoptotic agents that can sensitize pancreatic ductal adenocarcinoma cells to TRAIL and thereby overcome pancreatic cancer resistance to apoptosis. One intriguing finding that has been shown consistently by our group and others is the selective killing of pancreatic cancer cells by VEDT [5, 16, 19–21]. We have also shown selective induction of apoptosis by VEDT of pancreatic-transformed and malignant epithelial cells but not normal immortalized human pancreatic ductal epithelial cells [16, 19]. Additionally, our animal studies showed that VEDT not only caused a statistically significant inhibition of tumor growth but also resulted in apoptosis of tumor tissues [16, 20, 21]. These results clearly demonstrate that induction of apoptosis is implicated in the antitumor activity of VEDT.

In the current study, we found that VEDT alone triggered a caspase-8-dependent apoptosis in pancreatic cancer cells; however, when combined with TRAIL, VEDT significantly augmented and potentiated the TRAIL-induced apoptosis of pancreatic cancer cells. Specifically, we were able to demonstrate in vitro and in vivo increased cleaved caspase-8 and caspase-3 and induction of apoptotic cell death with VEDT treatment versus controls. Conversely, pharmacological inhibition of caspase-8 and caspase-3 demonstrated significant attenuation of VEDT-induced apoptosis. Together, these results strongly suggest that VEDT induces apoptosis through activation of the caspase-8 cascade. The role of caspase-8 in death receptor-mediated apoptosis has been extensively studied [24, 32, 33]. Interestingly, whereas pancreatic cancer cells have been shown to express all of the proteins of the extrinsic apoptotic pathway, most cell lines remain relatively insensitive to TRAIL-induced apoptosis. Both recombinant and antibody-based TRAIL therapies have been developed to date for the treatment of many cancers, but resistance to death receptor-mediated apoptosis remains a significant barrier in the field of pancreatic cancer [34].

A possible molecular target for VEDT is c-FLIP_s_, an inhibitor of caspase-8 in pancreatic cancer cells. We had shown that VEDT but not tocopherols augment TRAIL activity (unpublished data). Our data show that VEDT decreased c-FLIP_s_ levels without consistently modulating the expression of decoy death receptors 1, 2, and 3 or death receptors 4 and 5. Furthermore, VEDT was found to inhibit c-FLIP_s_ expression and induce caspase-8-dependent apoptosis in pancreatic cancer cells, followed by caspase-3 and PARP1 cleavage in a time-dependent manner. The activation of caspases-8 and -3 were required for VEDT induction of apoptosis, as demonstrated by rescue with specific caspase inhibitors. Moreover, the growth of pancreatic xenograft tumors in mice was significantly inhibited when treated with VEDT. This inhibition was also accompanied by an induction of apoptosis in tumors and depletion of c-FLIP_s_ protein expression. Although VEDT inhibited c-FLIP_s_ protein levels by 6 h, mRNA levels were actually increased at this time point, suggesting that VEDT may be inhibiting c-FLIP_s_ through protein degradation. From these analyses, we concluded that the mechanism of c-FLIP_s_ down-regulation is unlikely caused by transcriptional repression but rather an ubiquitin-mediated, proteasome-dependent process. Moreover, ferroptosis is a newly discovered type of cell death that differs from traditional apoptosis and necrosis and results from iron-dependent lipid peroxide accumulation and autophagy [35, 36]. Pancreatic ductal adenocarcinoma with a mutant *KRas* gene is more susceptible to ferroptosis, and it might be related to the tumorigenesis of pancreatic carcinoma [37]. Therefore, VEDT may kill pancreatic cancer cells through ferroptosis. This possibility will be investigated in our future studies.

## Conclusions

We showed that VEDT decreased c-FLIP levels by promoting ubiquitin/proteasome-mediated degradation of c-FLIP. In summary, our data indicate that VEDT down-regulates c-FLIP_s_, an inhibitor of caspase-8 activation through protein degradation, and induces apoptosis through activation of caspase-8 and caspase-3. This works suggests that VEDT should be evaluated for targeting programed cell death in pancreatic cancer cells.

## Additional files

**Additional file 1: Fig. S1.** Chemical structures of vitamin E analogs and effect of 8 members of the vitamin E family on cell survival in MiaPaCa-2 cells. **(A)** Chemical structures of the vitamin E analogs. **(B)** Effect of the 8 members of the vitamin E family on cell survival in MiaPaCa-2 cells. Points, means; bars, standard error (n = 3-5, *P < .001, **P < .01). **(C)** Effect of the 8 members of the vitamin E family on c-FLIP expression in MiaPaCa-2 cells (n = 3).

**Additional file 2: Fig. S2.** Effects of VEDT and TRAIL on cell death and Western blot analyses. **(A)** Effect of VEDT (50 µM) and TRAIL (25 ng/mL) alone and in combination on cell death (Trypan blue) of immortalized human pancreatic normal epithelial (HPNE-vector) cells and HPNE-Kras cells. VEDT, TRAIL, or the combination of the 2 drugs did not cause significant cell death in HPNE-vector cells (n = 3-5). VEDT and TRAIL alone significantly induced cell death compared to vehicle (^a^*P *< .02 and ^b^*P *< .05, respectively) in HPNE-Kras cells (n = 3-5). However, greater significant cell death occurred when agents were combined than with vehicle (^c^*P *< .01) and either drug alone (^d^*P *< .05). **(B)** Western blot analyses of endogenous and exogenous c-FLIP_s_ protein expression in MiaPaCa-2 cells. Mock transfection and pCMV6-AC-GFP vector transfections served as internal controls, whereas β-actin served as loading control. c-FLIP_s_ expression is shown in parental MiaPaCa-2 cells and in MiaPaCa-2 cells stably expressing pCMV6-AC-GFP vector alone (Mia-GFP) or containing c-FLIP_s_ (Mia-FLIP) (n = 3). **(C)** VEDT inhibited c-FLIP_s_ expression in Mia-GFP cells compared to vehicle (V) after 24 h and the expression was rescued in Mia-FLIP cells (n = 3). **(D)** VEDT (T) induced apoptosis (PARP cleavage) in (Mia-GFP) cells compared to vehicle (V) after 24 h. CF = cleaved fragment (n = 3) and **(E)** Immunofluorescence staining of apoptosis (TUNEL) show that VEDT induced apoptosis in (Mia-GFP) cells compared to vehicle (Veh) after 24 h and apoptosis was rescued in (Mia-FLIP) cells compared to vehicle (Veh) (n = 3).

**Additional file 3: Fig. S3.**Effects of VEDT and TRAIL on apoptosis Effects of VEDT (50 µM) and TRAIL (25 ng/mL) alone and in combination on apoptosis (Annexin V/PI) of Panc-1 cells. VEDT and TRAIL induced apoptosis (25% and 23%, respectively) compared to vehicle in Panc-1 cells. However, greater apoptosis occurred when the 2 drugs were combined than occurred with vehicle alone (49%) in Panc-1 cells.

## Data Availability

Data will be made available upon reasonable request.

## Abbreviations

VEDT: Vitamin E delta-tocotrienol
c-FLIP: cellular FLICE inhibitory protein
GFP: green fluorescence protein
HPNE: human pancreatic normal epithelial
TUNEL: terminal deoxynucleotidyl transferase-mediated nick end labeling
PBS: phosphate-buffered saline
IP: intraperitoneal
ANOVA: analysis of variance
PARP: poly ADP ribose polymerase
TRAIL: tumor necrosis factor-related apoptosis-inducing ligand
FLIP: FLICE inhibitory protein
HA: hemagglutinin
SRB: sulforhodamine B
