# Supplementary material for: Vitamin E δ-tocotrienol sensitizes human pancreatic cancer cells to TRAIL-induced apoptosis through proteasome-mediated down-regulation of c-FLIPs
Source: Cancer Cell Int. 2019 Jul 22;19:189. doi: 10.1186/s12935-019-0876-0 (PMC6647259; doi:10.1186/s12935-019-0876-0)
Supplement: Supplementary file 2 — Additional file 2: Fig. S2. Effects of VEDT and TRAIL on cell death and Western blot analyses. (A) Effect of VEDT (50 µM) and TRAIL (25 ng/mL) alone and in combination on cell death (Trypan blue) of immortalized human pancreatic normal epithelial (HPNE-vector) cells and HPNE-Kras cells. VEDT, TRAIL, or the combination of the 2 drugs did not cause significant cell death in HPNE-vector cells (n = 3-5). VEDT and TRAIL alone significantly induced cell death compared to vehicle (aP < .02 and bP < .05, respectively) in HPNE-Kras cells (n = 3-5). However, greater significant cell death occurred when agents were combined than with vehicle (cP < .01) and either drug alone (dP < .05). (B) Western blot analyses of endogenous and exogenous c-FLIPs protein expression in MiaPaCa-2 cells. Mock transfection and pCMV6-AC-GFP vector transfections served as internal controls, whereas β-actin served as loading control. c-FLIPs expression is shown in parental MiaPaCa-2 cells and in MiaPaCa-2 cells stably expressing pCMV6-AC-GFP vector alone (Mia-GFP) or containing c-FLIPs (Mia-FLIP) (n = 3). (C) VEDT inhibited c-FLIPs expression in Mia-GFP cells compared to vehicle (V) after 24 h and the expression was rescued in Mia-FLIP cells (n = 3). (D) VEDT (T) induced apoptosis (PARP cleavage) in (Mia-GFP) cells compared to vehicle (V) after 24 h. CF = cleaved fragment (n = 3) and (E) Immunofluorescence staining of apoptosis (TUNEL) show that VEDT induced apoptosis in (Mia-GFP) cells compared to vehicle (Veh) after 24 h and apoptosis was rescued in (Mia-FLIP) cells compared to vehicle (Veh) (n = 3). [file 12935_2019_876_MOESM2_ESM.docx]

**Supplementary Figure S2**

Effects of VEDT and TRAIL on cell death and Western blot analyses


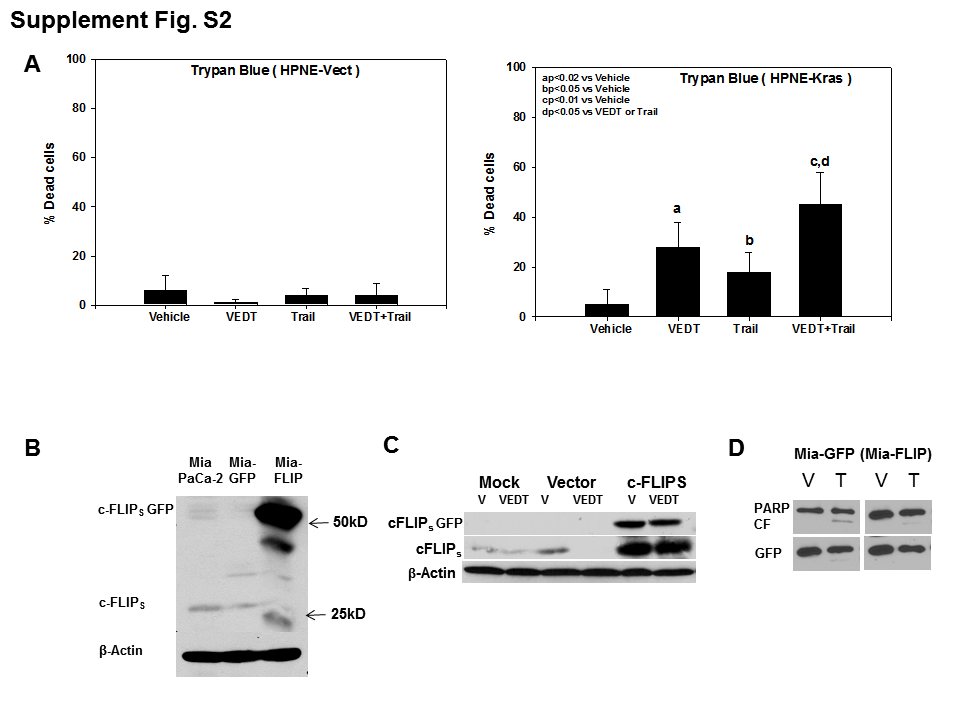

**Figure S2**

**(A)** Effect of VEDT (50 µM) and TRAIL (25 ng/mL) alone and in combination on cell death (Trypan blue) of immortalized human pancreatic normal epithelial (HPNE-vector) cells and HPNE-Kras cells. VEDT, TRAIL, or the combination of the 2 drugs did not cause significant cell death in HPNE-vector cells (n = 3-5). VEDT and TRAIL alone significantly induced cell death compared to vehicle (^a^*P* < .02 and ^b^*P* < .05, respectively) in HPNE-Kras cells (n = 3-5). However, greater significant cell death occurred when agents were combined than with vehicle (^c^*P* < .01) and either drug alone (^d^*P* < .05). **(B)** Western blot analyses of endogenous and exogenous c-FLIP_s_ protein expression in MiaPaCa-2 cells. Mock transfection and pCMV6-AC-GFP vector transfections served as internal controls, whereas β-actin served as loading control. c-FLIP_s_ expression is shown in parental MiaPaCa-2 cells and in MiaPaCa-2 cells stably expressing pCMV6-AC-GFP vector alone (Mia-GFP) or containing c-FLIP_s_ (Mia-FLIP) (n = 3). **(C)** VEDT inhibited c-FLIP_s_ expression in Mia-GFP cells compared to vehicle (V) after 24 h and the expression was rescued in Mia-FLIP cells (n = 3). **(D)** VEDT (T) induced apoptosis (PARP cleavage) in (Mia-GFP) cells compared to vehicle (V) after 24 h.CF= cleaved fragment (n = 3) and **(E)** Immunofluorescence staining of apoptosis (TUNEL) show that VEDT induced apoptosis in (Mia-GFP) cells compared to vehicle (Veh) after 24 h and apoptosis was rescued in (Mia-FLIP) cells compared to vehicle (Veh) (n = 3).
