# Supplementary material for: Vitamin E δ-tocotrienol sensitizes human pancreatic cancer cells to TRAIL-induced apoptosis through proteasome-mediated down-regulation of c-FLIPs
Source: Cancer Cell Int. 2019 Jul 22;19:189. doi: 10.1186/s12935-019-0876-0 (PMC6647259; doi:10.1186/s12935-019-0876-0)
Supplement: Supplementary file 3 — Additional file 3: Fig. S3.Effects of VEDT and TRAIL on apoptosis Effects of VEDT (50 µM) and TRAIL (25 ng/mL) alone and in combination on apoptosis (Annexin V/PI) of Panc-1 cells. VEDT and TRAIL induced apoptosis (25% and 23%, respectively) compared to vehicle in Panc-1 cells. However, greater apoptosis occurred when the 2 drugs were combined than occurred with vehicle alone (49%) in Panc-1 cells. [file 12935_2019_876_MOESM3_ESM.docx]

**Supplementary Figure S3**

Chemical structures of vitamin E analogs and effect of 8 members of the vitamin E family on cell survival in MiaPaCa-2 cells.

**
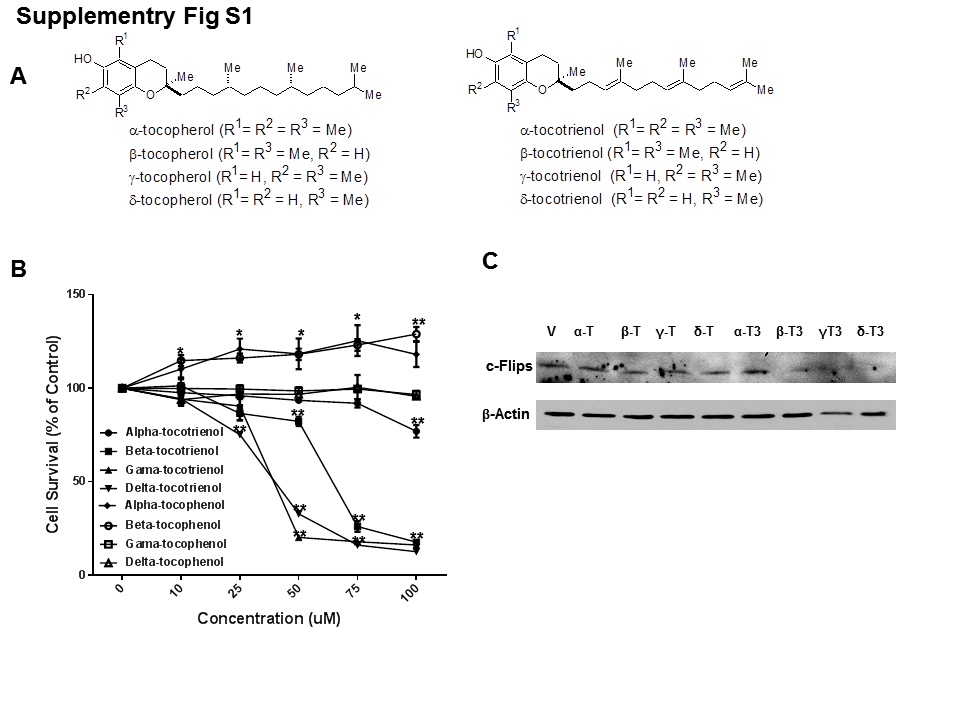
**

**(A)** Chemical structures of the vitamin E analogs. **(B)** Effect of the 8 members of the vitamin E family on cell survival in MiaPaCa-2 cells. Points, means; bars, standard error (n = 3-5, **P* < .001, ***P* < .01). **(C)** Effect of the 8 members of the vitamin E family on c-FLIP expression in MiaPaCa-2 cells (n = 3).
